# A_3_ Adenosine Receptor Antagonists with Nucleoside Structures and Their Anticancer Activity

**DOI:** 10.3390/ph15020164

**Published:** 2022-01-28

**Authors:** Andrea Spinaci, Michela Buccioni, Diego Dal Ben, Federica Maggi, Gabriella Marucci, Beatrice Francucci, Giorgio Santoni, Catia Lambertucci, Rosaria Volpini

**Affiliations:** 1 Medicinal Chemistry Unit, School of Pharmacy, University of Camerino, 62032 Camerino, MC, Italy; andrea.spinaci@unicam.it (A.S.); michela.buccioni@unicam.it (M.B.); diego.dalben@unicam.it (D.D.B.); gabriella.marucci@unicam.it (G.M.); beatrice.francucci@unicam.it (B.F.); 2 Experimental Medicine Section, School of Pharmacy, University of Camerino, 62032 Camerino, MC, Italy; federica.maggi@uniroma1.it (F.M.); giorgio.santoni@unicam.it (G.S.); 3 Department of Molecular Medicine, Sapienza University, 00185 Rome, RM, Italy

**Keywords:** A_3_ adenosine receptors, A_3_ adenosine receptor antagonists, adenosine derivatives, anticancer activity, sulforhodamine B assay

## Abstract

The overexpression of the A_3_ adenosine receptor (AR) in a number of cancer cell types makes it an attractive target for tumor diagnosis and therapy. Hence, in the search for new A_3_AR ligands, a series of novel 2,*N*^6^-disubstituted adenosines (Ados) was synthesized and tested in radioligand binding and functional assays at ARs. Derivatives bearing a 2-phenethylamino group in the *N*^6^-position were found to exert higher A_3_AR affinity and selectivity than the corresponding *N*^6^-(2,2-diphenylethyl) analogues. 2-Chloro-*N*^6^-phenylethylAdo (**15**) was found to be a potent full A_3_AR agonist with a Ki of 0.024 nM and an EC_50_ of 14 nM, in a cAMP accumulation assay. Unlike **15**, the other ligands behaved as A_3_AR antagonists, which concentration-dependently reduced cell growth and exerted cytostatic activity on the prostate cancer cell line PC3, showing comparable and even more pronounced effects with respect to the ones elicited by the reference full agonist Cl-IB-MECA. In particular, the *N*^6^-(2,2-diphenylethyl)-2-phenylethynylAdo (**12**: GI_50_ = 14 µM, TGI = 29 µM, and LC_50_ = 59 µM) showed the highest activity proving to be a potential antitumor agent. The cytostatic effect of both A_3_AR agonist (Cl-IB-MECA) and antagonists (**12** and other newly synthesized compounds) confirm previous observations according to which, in addition to the involvement of A_3_ARs, other cellular mechanisms are responsible for the anticancer effects of these ligands.

## 1. Introduction

**Figure 1:**
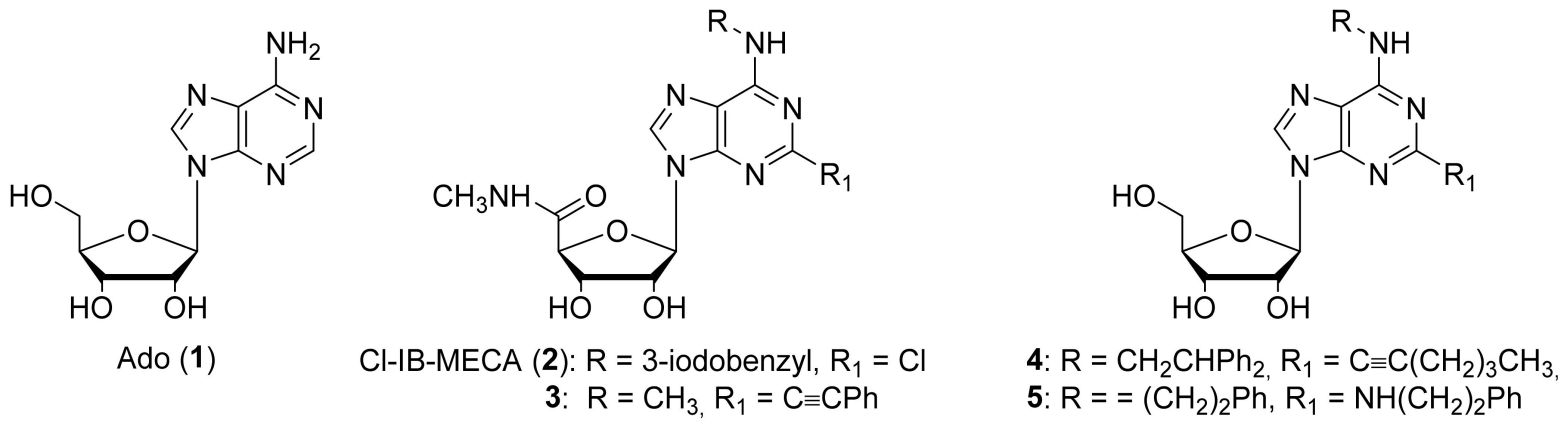
Structure of known A_3_AR ligands: Ado (**1**), Cl-IBMECA (**2**), **3–5**.

Ubiquitous nucleosides and nucleotides are involved in several biological functions and they play a crucial role in some mechanisms, such as cell growth, migration, differentiation, bacterial-induced inflammation and growth factor secretion [1,2,3,4]. The nucleoside adenosine (Ado, **1**, Figure 1) performs its function by interacting with the four A_1_, A_2A_, A_2B_ and A_3_ adenosine receptors (ARs) belonging to the superfamily of G protein-coupled receptors (GPCRs). The activation of A_1_AR and A_3_AR mainly produces a decrease of the intracellular cyclic adenosine monophosphate (cAMP) concentration, while stimulation of the A_2A_AR and A_2B_AR leads to an opposite effect. 

In physiological conditions, this peculiar nucleoside is present in almost all cells and tissues in low concentrations (nM range), while in stress conditions such as hypoxia, which characterizes tumors, its concentration increases (µM range) [2]. A_3_ARs overexpression in various types of cancer cell has been reported in numerous studies [5] and has been demonstrated in lung, breast, melanoma, prostate, pancreatic, and liver cancers, as well as malignant pleural mesothelioma (MPM), glioblastoma, and lymphoma [2,6,7]. 

However, the particular issue is focused on the role of the A_3_AR in regulating cell proliferation and death, as this receptor acts differently depending on the type of tissue in which it is expressed [8]. On this basis, the A_3_AR is an attractive target for cancer diagnosis or for its ability to counteract the tumor grow, although there is evidence that, in some cancer types, activation of the A_3_AR promotes cell proliferation and survival, while in others it activates cytostatic and apoptotic pathways [9,10]. 

This dual behavior seems to be due to the different regulation/dysregulation of the Wnt pathway induced by A_3_AR activation in different tumor types [10]. In this regard, the mechanisms that influence the cancer cells’ proliferation or death could also be due to the modulation of Ado levels, which can be modified by affecting the pathways of Ado generation, degradation, and elimination [11,12]. Furthermore, A_3_AR ligands conjugated with metallacarborans, containing iron and cobalt, protect cells from cross-resistance showed by anticancer drugs such as cisplatin, carboplatin, and doxorubicin [13,14]. 

The known full A_3_AR agonist, 2-chloro-*N*^6^-(3-iodobenzyl)adenosine-5′-*N*-methyluronamide (Cl-IB-MECA, also called CF-102 or Namodenoson, **2**, Figure 1), has been extensively studied on tumor cell proliferation and this compound has been reported to inhibit cancer cell proliferation in in vitro and in vivo tumor models [15,16]. In a randomized, placebo-controlled phase II trial for hepatocellular carcinoma and moderate hepatic dysfunction, Cl-IB-MECA did not meet its primary end-point even though it showed a favorable safety profile and preliminary efficacy [17]. 

Recently, we reported the ability of Cl-IB-MECA analogues and Ado derivatives to inhibit cell growth of human prostate (PC3), colon (Caco-2) and hepatocyte (Hep G2) carcinomas, using Cl-IB-MECA as the reference compound [18]. The results showed a good anticancer effect in all three tumor cell lines by Cl-IB-MECA, while its analogue *N*^6^-methyl-2-phenylethynyladenosine-5′-*N*-methyluronamide (**3**, Figure 1) was almost inactive. On the contrary, a very good antitumor activity, comparable to that of Cl-IB-MECA itself, was exerted by the two potent and selective A_3_AR ligands, *N*^6^-(2,2-diphenylethyl)-2-hexynylAdo (**4**) and *N*^6^-(2-phenylethyl)-2-(2-phenylethylamino)Ado (**5**), which are Ado derivatives, bearing steric hindering aromatic groups in *N*^6^- and 2-positions, the synthesis of which has not yet been published (Figure 1) [18]. 

It is worthwhile to note that compound **3** behaves as a full A_3_AR agonist endowed with higher A_3_AR affinity and selectivity compared to Cl-IB-MECA [18,19]. The lack of anticancer activity of compound **3** indicated that the active compounds exerted their cytotoxic activity not only through the interaction with A_3_ARs and suggested the possible involvement of other cellular mechanisms. Indeed, further experiments led to hypothesize that the anticancer activity of compound **4** was due to its ability to induce apoptosis and to raise the level of reactive oxygen species (ROS) [18]. 

**Figure 2:**
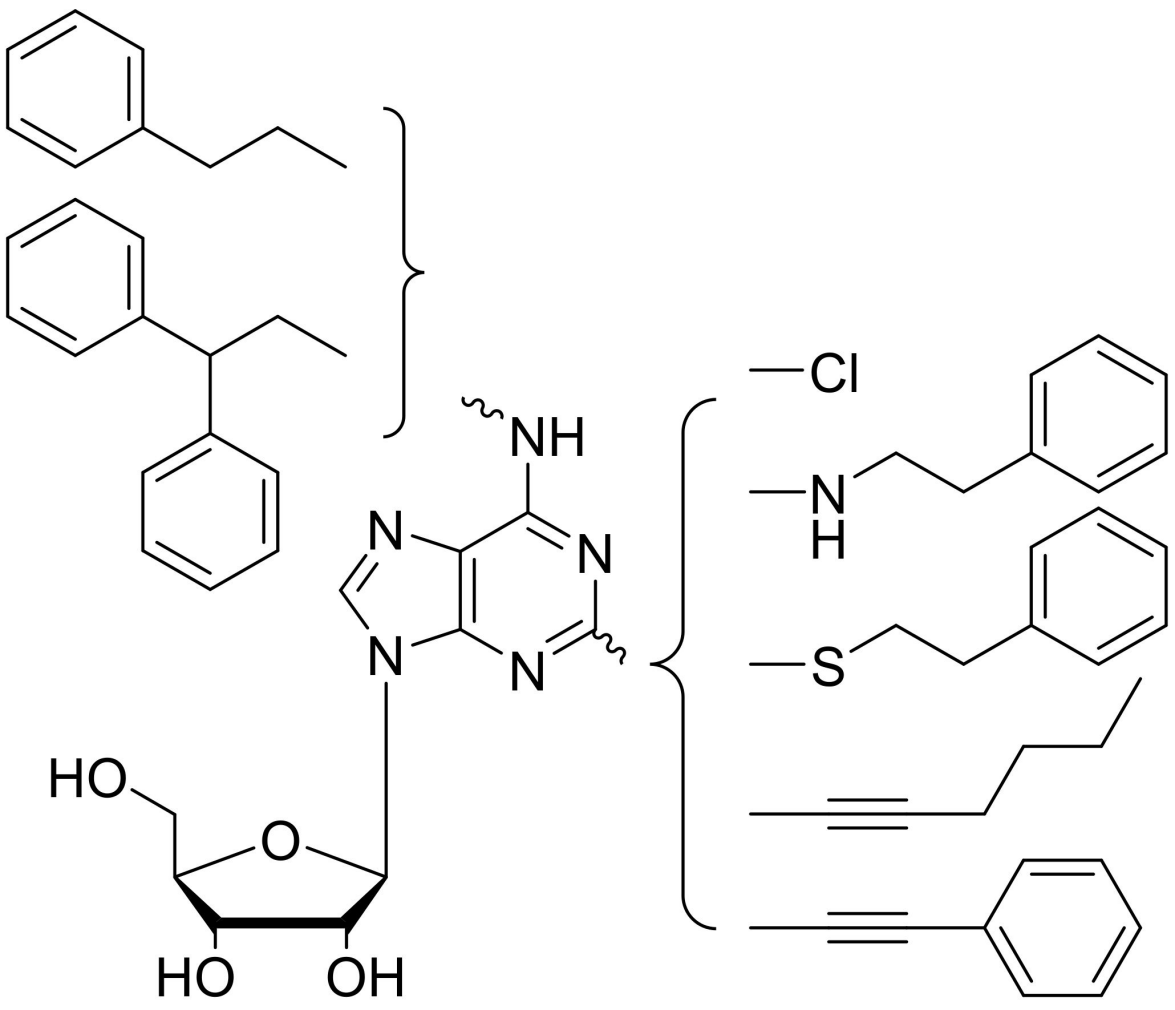
General structure of synthesized compounds.

Starting from these observations, the purpose of this work was to identify new potential anticancer agents and to verify that the antitumor activity of these molecules is not closely associated with the A_3_AR binding affinity, confirming the possible intervention of other mechanisms. Hence, taking into account the structure of the two Ado derivatives **4** and **5**, which showed cytotoxic activity in the three tumor cell lines mentioned above, in the present work, the synthesis of a new series of 2,*N*^6^ di-substituted Ado derivatives bearing the same substituents of **4** and **5** at the *N*^6^-position and a chlorine atom, different alkynyl chains and a phenethylthio group at the 2-position, was undertaken (Figure 2).

The new compounds were tested in radioligand binding and functional assays, to assess their affinity (A_1_, A_2A_, and A_3_ARs) and potency (A_2B_AR) at human ARs. Furthermore, they were tested in a PC3 prostate cell line, to establish their antiproliferative and cytotoxic activities, and in a functional cAMP assay to evaluate their ability to activate the A_3_AR. Since the lack of sugar modification can lead to A_3_AR antagonists [19], this last experiment was aimed at verifying whether these compounds are able or not to activate the A_3_AR and, therefore, if they behave like Cl-IB-MECA.

## 2. Results and Discussion

### 2.1. Chemistry

**Scheme 1:**
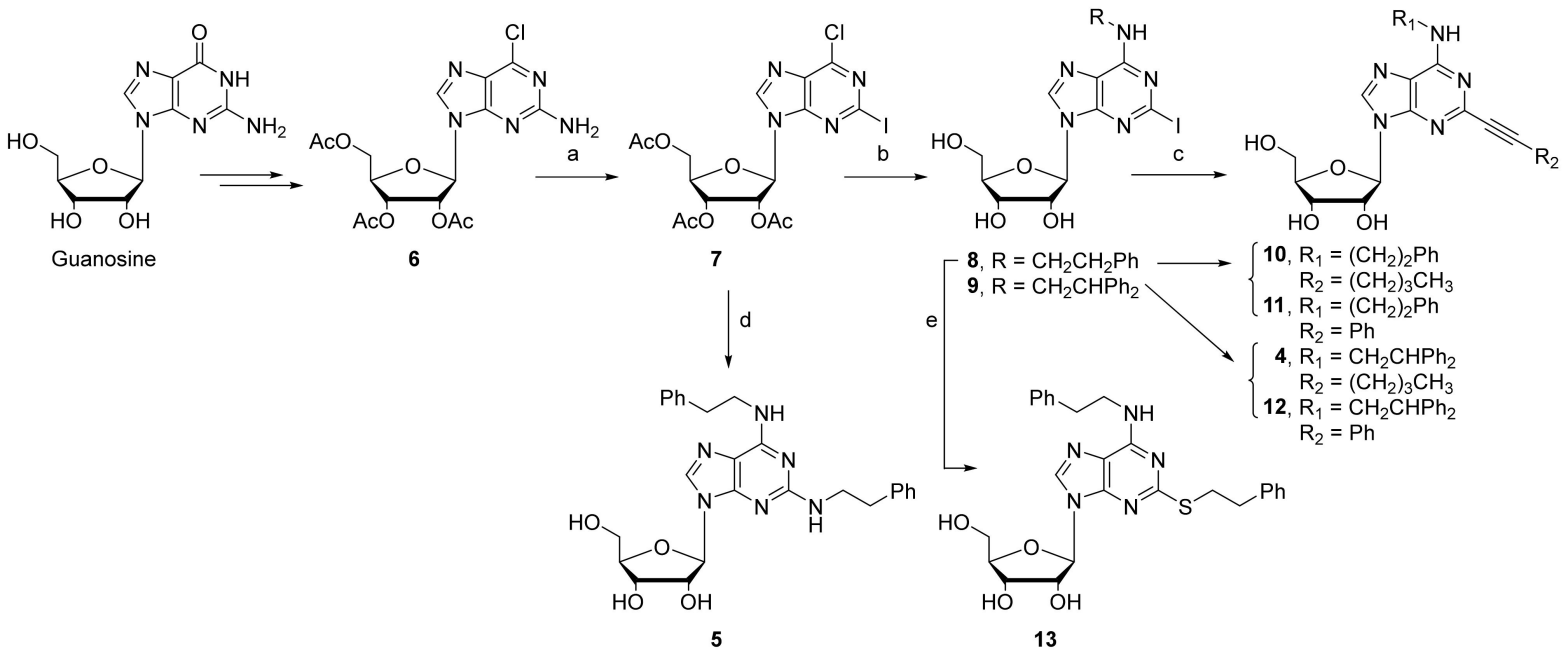
Reagents and conditions: (a) C_5_H_11_ONO, CH_2_I_2_, THF, 65 °C; (b) 1. Ph(CH_2_)_2_NH_2_, K_2_CO_3_, DMF, r.t., 16 h; 2. NH_3_/CH_3_OH, r.t. 30 min, 67% yield; or 1. Ph_2_CHCH_2_NH_2_, Et_3_N, CH_3_CN, r.t. 8 hs; 1. NH_3_/CH_3_OH, r.t. 1 h, 98% yield; (c) R-C≡CH, (Ph_3_P)_2_PdCl_2_, CuI, Et_3_N, DMF, r.t, 16 h, 38–70% yield.; (d) 1. Ph(CH_2_)_2_NH_2_, K_2_CO_3_, DMF, 120 °C, 16 h; 2. NH_3_/CH_3_OH, r.t. 30 min, 81% yield; (e) Ph(CH_2_)_2_SH, K_2_CO_3_, DMF, 120 °C, 16 h, 75% yield.

**Scheme 2:**
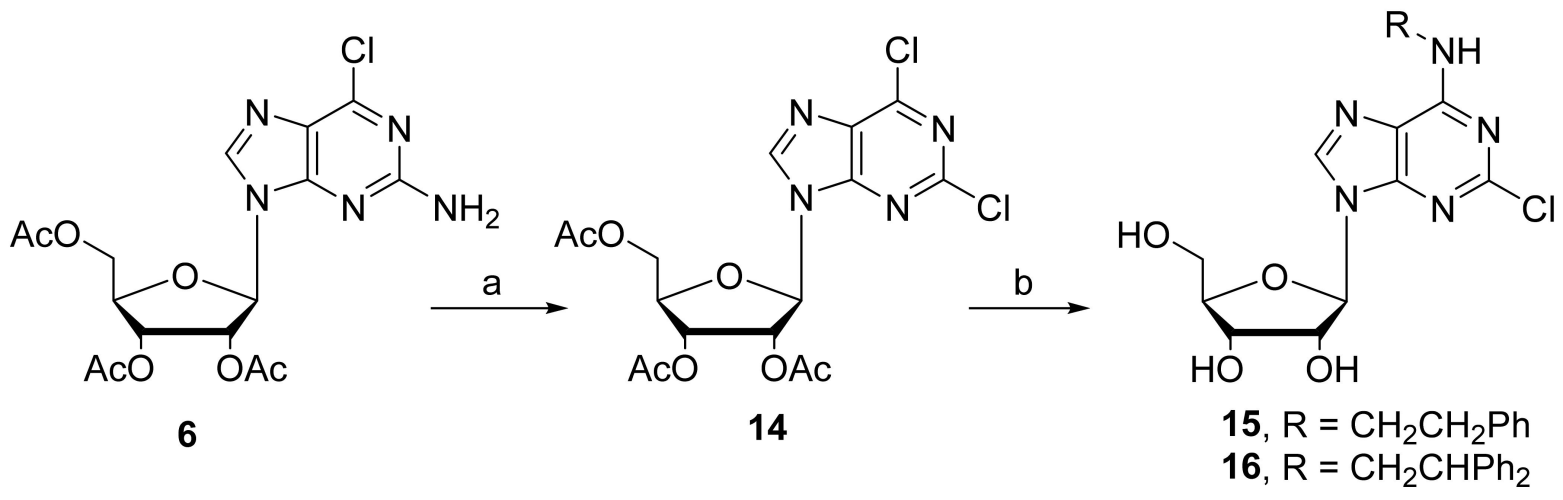
Reagents and conditions: (a) C_5_H_11_ONO, SbCl_3_, CH_2_Cl_2_, 0 °C, 5 h, 73% yield; (b) 1. R-NH_2_, Et_3_N, EtOH, 0–25 °C, 15 h, 2. NH_3_/MEOH, r.t., 5 h; 47–67% yield.

Here, we report the synthesis of the newly nucleosides **10–13**, **15**, and **16**, together with that of the previously reported compounds **4** and **5**, which was carried out using a divergent approach starting from commercial guanosine (Scheme 1 and Scheme 2). 

Commercial guanosine was converted in the protected 2-amino-6-chloropurineriboside **6**, in two steps, as already described (Scheme 1) [20]. By a modification of the Sandmeyer reaction, consisting in a diazotization with isoamyl nitrite and diiodomethane, the 2-iodo derivative **7** was prepared from **6**, using dry THF as a solvent, without iodine as previously reported [21]. The 6-chloro-2-iodopurineriboside **7** was then reacted with 2-phenethylamine or 2,2-diphenylethylamine, at r. t., to selectively displace the 6-chlorine atom. Then, methanolic ammonia was added at r. t. for the complete removal of protecting groups at the sugar moiety. The corresponding 2-iodo-*N*^6^-substituted Ados **8** and **9** were obtained as white powders after chromatography, with very good yields (Scheme 1). 

The reaction of **7** to give 2-phenethylamino-*N*^6^-phenethylAdo (**5**) was performed in two steps. First, **7** was reacted with phenethylamine at 120 °C in a sealed vial, using DMF and potassium carbonate as a solvent and catalyst, respectively. Since these conditions led to the substitution of both the halogens in the 2- and 6-positions and the partial deprotection of the sugar moiety, the mixture, in a second step, was treated with methanolic ammonia at r. t. to have the complete sugar deprotection and to obtain **8** (Scheme 2).

Reaction of 2-iodo-*N*^6^-phenylethylAdo (**8**) with phenylethylthiol, in dry DMF and in the presence of potassium carbonate at 120 °C, in a steel vial, furnished the 2-thioderivative **13**, with good yield after chromatography (Scheme 1). 

The 2-alkynylderivatives **10–12** and **4** were prepared by reacting the intermediates **8** or **9** under Sonogashira cross-coupling conditions, using the suitable 1-alkyne and bis(triphenylphosphine)palladium dichloride, copper iodide, and triethylamine as catalysts, in DMF dry as solvent (Scheme 1). 

The 2-chloroadenosine derivatives **15** and **16** were synthesized starting from **6**, which was reacted with isoamyl nitrite and antimony chloride in DCM, at 0 °C, to get the 2,6-dichloropurineriboside **14**. This compound was, in turn, treated with 2-phenethylamine and 2,2-diphenylethylamine, using ethanol as solvent and potassium carbonate as catalyst, to obtain the desired nucleosides **15** and **16** (Scheme 2).

### 2.2. Binding Assay at A_1_, A_2A_, and A_3_ ARs and Functional Studies at A_2B_ARs

The new compounds **10–13** and **15–16**, together with the 2-iodo nucleoside intermediates **8** and **9**, were tested in radioligand binding assay at human recombinant ARs, expressed in Chinese hamster ovary (CHO) cells, to evaluate their affinity for A_1_, A_2A_, and A_3_ AR subtypes. [^3^H]CCPA (2-chloro-*N*^6^-cyclopentylAdo), [^3^H]NECA (5′-*N*-ethylcarboxamidoAdo), and [^3^H]HEMADO (2-hexynyl-*N*^6^-methylAdo) were used as respective radioligands [22,23]. The results are reported as Ki values in nM (± standard errors) (Table 1). In the case of A_2B_AR, the potency of selected compounds was determined through a functional GloSensor cAMP assay [22]. Since their EC_50_ values resulted > 30 µM, these data are not shown in Table 1. 

Cl-IB-MECA and the already known A_3_AR ligands **4** and **5** are reported as reference compounds. Cl-IB-MECA is an A_3_AR full agonist endowed with high affinity (Ki = 1.4 nM) and a very good A_3_AR selectivity of 886 and 3829 fold vs. the A_1_ and A_2A_ ARs, respectively. The newly synthesized compounds behave, in general, as A_3_AR ligands with high affinity (Ki in the nM and sub-nM range) and various degrees of selectivity. In particular, the already reported 2-phenethylamino-*N*^6^-phenethylAdo (**5**) possesses a very high A_3_AR affinity with a KiA_3_AR = 0.33 nM and a selectivity of thousands of times versus both the other AR subtypes. This compound was the A_3_AR ligand endowed with the most balanced affinity and selectivity values for the A_3_AR, better also than the reference Cl-IB-MECA.

The replacement of the 2-phenethylamino group of **5** with halogens led to an increase of affinity compared to the A_1_AR subtype, but different results were found on the other subtypes. In fact, the 2-iodo derivative **8** showed a reduced affinity for both A_2A_ and A_3_ ARs, compared to **5**. On the contrary, for the 2-chloro derivative **15** there was an increase in affinity for these subtypes. It is worthwhile to note that the 2-chloro-*N*^6^-phenethylAdo (**15**) possesses an A_3_AR Ki in the pM range and a very high selectivity vs. the A_2A_ subtype (27,500 fold), although it is less selective for the A_1_AR with respect to the parent compound **5** and Cl-IB-MECA. 

Substitution of the phenethylamino group at the 2-position of **5** with different alkynyl chains, as in compounds **10** and **11**, or with the isosteric phenethylthio group, as in compound **13**, has resulted in derivatives that maintain high A_3_AR affinity, although with lower A_3_AR selectivity. The presence of a bulky 2,2-diphenylethyl group in the *N*^6^-position, as in compounds **9**, **16**, **4**, and **12**, caused a general decrease in A_3_AR affinity and selectivity (see **9** vs. **8**, **16** vs. **15**, **4** vs. **10**, and **12** vs. **11**). It should be noted that, also in this series of compounds, the 2-chloro derivative showed the highest A_3_AR affinity (**16**; KiA_3_AR = 0.13 nM).

### 2.3. Antiproliferative and Cytotoxic Assays

To assess the antitumor activity of all the synthesized compounds **4**, **5**, **8–13**, **15**, **16**, their antiproliferative and cytotoxic effects were evaluated in the PC3 cell line by the sulforhodamine B (SRB) assay, according to the National Cancer Institute protocol [25]. The reference A_3_AR agonist Cl-IB-MECA was tested with the same protocol for comparison, in order. The compounds were used at concentrations of 1, 10, 25, 50, and 100 μM for 48 h at 37 °C. The antitumor activity was estimated by measurements of three parameters: Growth Inhibition 50 (GI_50_), the compound concentration (µM) required to inhibit 50% net of cell growth; Total Growth Inhibition (TGI), the compound concentration (µM) required to inhibit 100% of cell growth; Lethal Concentration 50 (LC_50_), the compound concentration (µM) required to kill 50% of the initial cell number. The results are shown in Table 2 and Figure S1, along with A_3_AR affinity. 

The compounds demonstrated to concentration-dependently reduce cell growth of the PC3 prostate cancer cell line. Most of them showed a significant inhibitory effect on cell proliferation and a pronounced cytotoxic activity comparable to the one elicited by Cl-IB-MECA, after 48 h exposure. Compounds **5**, **8**, **13**, and **15**, bearing a phenethyl chain at the *N*^6^-position, exhibited a lower ability to inhibit cell proliferation and cell survival than Cl-IB-MECA, which showed a GI_50_ = 18 µM, TGI = 44 µM, and LC_50_ = 110 µM. On the contrary, compounds **10** and **11**, bearing the same *N*^6^-phenethyl group and an alkynyl chain at the 2-position, exhibited a higher cytostatic effect than Cl-IB-MECA (**10**: GI_50_ = 13 µM and **11**: GI_50_ = 2.5 µM and TGI = 19 µM). 

It should be noted that the *N*^6^-phenethyl-2-phenylethynylAdo (**11**) is endowed with the best cytostatic activity, among all the tested derivatives. Furthermore, two of these compounds, **13** and **15**, at a concentration of 500 µM, were not able to kill all the cells, in fact their LC_50_ was higher than 500 µM. It should be noted that, also in the series of the *N*^6^-(2,2-diphenylethyl) derivatives **4**, **9**, **12**, and **16**, the compounds bearing an alkynyl chain at the 2-position showed a pronounced cytostatic activity, being more active than Cl-IB-MECA. These two compounds were found to be more potent than the reference also for their cytotoxic effects (**4** and **12**: LC_50_ of 80 and 59 µM, respectively, compared to Cl-IB-MECA: LC_50_ = 110 µM). The *N*^6^-(2,2-diphenylethyl)-2-phenylethynylAdo (**12**) resulted as the most active compound with a GI_50_ = 14 µM, TGI = 29 µM, and LC_50_ = 59 µM. The observation that the A_3_AR ligands **5** (Ki = 0.33 nM), **15** (Ki = 0.024 nM), and **16** (Ki = 0.13 nM), were not those with the highest cytostatic and cytotoxic effects, despite their remarkable A_3_AR affinity, seems to demonstrate that the antitumor activity of the tested 2,*N*^6^-disubstitued Ados is not strictly correlated to their affinity for the A_3_AR subtype.

### 2.4. Functional Activity at Human A_3_AR

Among the four AR subtypes, A_3_AR appears to be the most sensitive to small chemical changes in its ligands. Indeed, various Ado derivatives, which were previously claimed as full or partial A_3_AR agonists, behaved subsequently as A_3_AR antagonists [24]. In several papers we have reported that the MECA and NECA derivatives substituted in 2-position with alkynyl chains and bearing a methyl or a methoxy group at the *N*^6^-position behave as full A_3_AR agonists like Cl-IB-MECA [19,26]. Conversely, the corresponding Ado derivatives lose efficacy, proving to act as partial agonists or antagonists on this receptor subtype [19,26]. Since the here presented newly synthesized A_3_AR ligands possess an intact ribose moiety in their structure, a functional assay was performed to measure their ability to activate the A_3_AR subtype. Then, the intrinsic activity of the selected compounds **4**, **10–12**, and **15**, chosen on the basis of their very high A_3_AR affinity or antitumor activity, was evaluated. In particular, the ligands were analyzed in a functional experiment to assess their ability to inhibit forskolin-stimulated cAMP production through the human A_3_AR, compared to the full agonist Cl-IBMECA [22].

Results showed that the reference compound Cl-IB-MECA and the 2-chloro-*N*^6^-phenethylAdo (**15**) were able to completely counteract the stimulation of adenylyl cyclase induced by 10 µM forskolin, so behaving as A_3_AR full agonists. On the contrary, compounds **4** and **10–12** did not affect cAMP levels induced by forskolin, when tested alone, but were able, to a different extent, to lower the effect of the full agonist, demonstrating behavior as A_3_AR antagonists. For all the compounds, the EC_50_ or IC_50_ values were calculated and the results are reported in Table 3 and Figure S2. The EC_50_ of Cl-IB-MECA was in a low nM range, according to the literature data [27], while the full agonist **15**, which showed the best A_3_AR affinity (KiA_3_AR = 0.024 nM), exhibited an EC_50_ of 14 nM. Among the four antagonists, **10** (IC_50_ = 31 nM) and **11** (IC_50_ = 79 nM), bearing a phenethyl group at the *N*^6^-position, showed a greater potency than **4** (IC_50_ = 380 nM) and **12** (IC_50_ = 153 nM), which present a 2,2-diphenylethyl chain in the same position. This order of potency was in close agreement with the compounds’ A_3_AR binding affinities. These data confirm that the efficacy of the A_3_AR ligands is closely related to the nature of the substituents present at the different positions of the nucleoside structure [19]. It is worth noting that the presence of a chlorine atom in 2-position of *N*^6^-phenethylAdo furnished the full A_3_AR agonist **15** like Cl-IB-MECA but, unlike the latter, **15** showed no cytotoxic activity at concentrations up to 500 µM. On the other hand, compounds **4** and **10–12**, which behaved as A_3_AR antagonists, demonstrated comparable and even greater antitumor activity than Cl-IB-MECA in PC3 prostate cells, at least in the cAMP accumulation assay. These inconsistencies, due to the antitumor activity of both the full agonist Cl-IB-MECA and the A_3_AR antagonists **4** and **10–12** on the same tumor cell type, cannot be explained by the different role played by the A_3_AR in the regulation of cell proliferation and death, depending on the tissue type in which it is expressed, but they suggest the possible involvement of other cellular mechanisms, as we have previously hypothesized [18]. On the other hand, this is not the first report in which A_3_AR antagonists have been found to exert anticancer activity [28,29]. 

## 3. Materials and Methods 

### 3.1. Chemical Synthesis

#### 3.1.1. General Methods

Melting points were determined with a Büchi apparatus and are uncorrected. ^1^H NMR spectra were obtained with a Bruker Ascend 500 MHz spectrometer; δ values are in ppm, J values are in Hz. All exchangeable protons were confirmed by the addition of D_2_O. Mass spectra were recorded on an HP 1100-MSD series instrument. Thin-layer chromatography (TLC) was carried out on pre-coated TLC plates with silica gel 60 F_254_ (Fluka). For column chromatography, silica gel 60 (Merck) was used. Elemental analyses were determined on Fisons Instruments Model EA 1108 CHNS-O model analyzer and are within 0.4% of theoretical values. Purity of the compounds is ≥98%, according to elemental analysis data.

#### 3.1.2. Synthesis of 6-Chloro-2-iodo-2′,3′,5′-tri-O-acetyladenosine (**7**)

Compound **6** (2.0 g, 4.68 mmol) was dissolved in anhydrous THF (5 mL), and diiodomethane (5.7 mL, 70.20 mmol), copper iodide (935 mg, 91 mmol) and isoamyl nitrite (2 mL, 14.51 mmol) were added. The mixture was refluxed for 5 h at 70 °C. The solvent was evaporated to dryness and the mixture was made into a slurry and purified by flash chromatography eluting with CHCl_3_-c-Hex (70:30) to obtain **7** as a white solid after recrystallization with MeOH. Yield: 79%; m.p.: 181–183 °C. ^1^H-NMR (DMSO-d6) δ: 1.99 (s, 3H, CH_3_), 2.04 (s, 3H, CH_3_), 2.09 (s, 3H, CH_3_), 4.27 (m, 2H, CH_2_-5′), 4.39 (m, 1H, H-4′), 5.60 (t, 1H, J = 11.0 Hz, H-3′), 5.86 (t, 1H, J = 10 Hz, H-2′), 6.27 (d, 1H, J = 5.2 Hz, H-1′), 8.80 (s, 1H, H-8). ESI-MS: positive mode m/z 539.0 [M+H]^+^, 561.0 [M+Na]^+^.

#### 3.1.3. Synthesis of 2-Iodo-*N*^6^-(2-phenylethyl)adenosine (**8**)

2-phenylethylamine (0.49 mL, 3.89 mmol) and K_2_CO_3_ (2.56 g, 18.55 mmol) were added to a solution of **7** (2.0 g, 3.71 mmol) in dry DMF (19 mL), and the mixture was left to react for 16 h under nitrogen atmosphere at r.t. After that, methanol saturated by ammonia (NH_3_/MeOH, 10 mL) was added and the mixture left under stirring for 30 min. After the removal of all volatiles under vacuum, the crude was purified by flash column chromatography eluting with DCM-MeOH (100% DCM to 95:5) and recrystallization from DCM/n-Hex mixture to obtain **8** as a white powder. Yield: 67%, m.p.: 120–122 °C. ^1^H-NMR (DMSO-d_6_) δ: 3.67 (m, 2H, CH_2_Ph) 4.35 (m, 1H, HCH-5′), 4.40 (m, 1H, HCH-5′), 4.44 (m, 2H, NHCH_2_), 4.71 (q, 1H, J = 3.6 Hz, H-4′), 4.9 (q, 1H, J = 3.2 Hz, H-3′), 5.3 (m, 1H, H-2′), 5.84 (d, 1H, J = 4.4 Hz, OH), 6.01 (m, 1H, OH), 6.26 (d, 1H, J = 6.4 Hz, OH), 6.59 (d, 1H, J = 6.0 Hz, H-1′), 7.98 (m, 1H, H-Ph), 8.08 (m, 5H, H-Ph and N^6^-H), 9.10 (s, 1H, H-8). ESI-MS: positive mode m/z 598.3 [M+H]^+^, 520.3 [M+Na]^+^. Anal (C_18_H_20_IN_5_O_4_) C, H, N.

#### 3.1.4. Synthesis of 2-Iodo-*N*^6^-(2,2-diphenylethyl)adenosine (**9**)

Compound **7** was suspended in CH_3_CN (4 mL) and 2,2-diphenylethylamine (0.62 mmol, 122 mg) was added, followed by Et_3_N (2.24 mmol, 312 µL). The solution was stirred at r. t. for 8 hrs. NH_3_/MeOH (5 mL) was added to the mixture and stirred for 1 h at r. t. All volatiles were removed under vacuum and the crude was made into slurry, the mixture was chromatographed by flash column eluting with CHCl_3_-MeOH (99:1–96:4) to obtain **9** as a white powder. Yield 98%, m.p.: 170–172 °C. ^1^H-NMR (DMSO-d6) δ: 3.51 (m, 1H, HCH-5′), 3.61 (m, 1H, HCH-5′), 3.89 (t, 1H, J = 6.0 Hz, H-4′), 4.01 (t, 2H, CH_2_-Ph) 4.08 (m, 2H, H-3′), 4.45 (m, 1H, H-2′), 4.55 (t, 1H, J = 6.2 Hz, NH-CH_2_), 5.00 (d, 1H, J = 5.0 Hz, OH), 5.18 (d, 1H, J = 5.0 Hz, OH), 5.45 (d, 1H, J = 5.0 Hz, OH), 5.77 (d, 1H, J = 5.0 Hz, H-1′), 7.17 (m, 6H, H-Ph), 7.29 (m, 4H, H-Ph), 8.22 (s, 1H, H-8), 8.26 (t, 1H, J = 5.5 Hz, NH). ESI-MS: positive mode m/z 574.0 [M+H]^+^, 596.0 [M+Na]^+^. Anal (C_24_H_24_IN_5_O_4_) C, H, N.

#### 3.1.5. 2-Phenylethylamino-*N*^6^-(2-phenylethyl)adenosine (**5**)

2-phenylethylamine (0.41 mL, 3.25 mmol) and K_2_CO_3_ (127.85 mg, 0.92 mmol) were added to a solution of **7** (100 mg, 0.185) in DMF in a sealed steel bomb, and set at 120 °C for 16 hrs. Then, the reaction was cooled to r.t. and NH_3_/CH_3_OH was added and the reaction left for 30 min under stirring. After the removal of volatiles, the crude mixture was purified by flash column chromatography eluting with DCM-MeOH (100% DCM to 96:4) and recrystallization from DCM/n-Hex to obtain **5** as a light-brown powder. Yield 81%, m.p.: 135–137 °C. ^1^H-NMR (DMSO-d_6_) δ: 2.87 (m, 4H, 2 x NHCH_2_CH_2_-Ph), 3.48 (m, 4H, 2 x NHCH_2_CH_2_Ph), 3.51 (m, 1H, HCH-5′), 3.63 (m, 1H, HCH-5′), 3.87 (q, 1H, J = 3.6 Hz, H-4′), 4.10 (q, 1H, J = 3.2 Hz, H-3′), 4.58 (m, 1H, H-2′), 5.10 (d, J = 4.4 Hz, 2H, 2 x OH), 5.36 (d, J = 6.4 Hz, 1H, OH), 5.73 (d, J = 6.0 Hz, 1H, H-1′), 6.32 (m, 1H, N^2^-H), 7.17 (m, 1H, H-Ph), 7.26 (m, 5H, H-Ph and N^6^-H), 7.89 (s, 1H, H-8). ESI-MS: positive mode m/z 490.9 [M+H]^+^, 512.8 [M+Na]^+^. Anal (C_26_H_30_N_6_O_4_) C, H, N.

#### 3.1.6. Synthesis of *N*^6^-(2-Phenylethyl)-2-phenylethylthioadenosine (**13**)

A mixture of **8** (395 mg, 0.24 mmol) in anhydrous DMF (6 mL), anhydrous K_2_CO_3_ (552 mg, 4 mmol), and 2-phenylethylthiol (500 μL, 4 mmol) was heated in a sealed steel bomb at 120 °C for 16 h. After removal of volatiles, the crude was purified via flash column chromatography eluting with CHCl_3_-MeOH (96:4) to obtain **13** as a white solid. Yield: 75%; m.p.: 126–128 °C; ^1^H-NMR (DMSO-d_6_) δ: 2.91 (m, 2H, PhCH_2_CH_2_NH), 2.98 (t, 2H, J = 7.6 Hz, SCH_2_CH_2_-Ph), 3.68 (m, 2H, NHCH_2_CH_2_Ph), 3.31 (m, 2H, SCH_2_), 3.53 (m, 1H, HCH-5′), 3.61 (m, 1H, HCH-5′), 3.92 (q, 1H, J = 4.0 Hz, H-4′), 4.11 (q, 1H, J = 4.0 Hz, H-3′), 4.55 (q, 1H, J = 6.0 Hz, H-2′), 5.07 (t, 1H, J = 5.2 Hz, OH), 5.19 (d, 1H, J = 4.8 Hz, OH), 5.43 (d, 1H, J = 6.0 Hz, OH), 5.84 (d, 1H, J = 6.4 Hz, H-1′), 7.20 (m, 2H, 2 x H-Ph), 7.30 (m, 8H, 2 x H-Ph), 8.05 (m, 1H, N^6^-H), 8.22 (s, 1H, H-8). ESI-MS: positive mode m/z 507.9 [M+H]^+^, 529.8 [M+Na]^+^, 1037.0 [2M+Na]^+^. Anal (C_26_H_29_N_5_O_4_S) C, H, N.

#### 3.1.7. General Procedure for the Synthesis of 2-Alkynyl-N^6^-substituted Adenosines **10**, **11**, **4**, and **12**

To a solution of **8** or **9** (0.37 mmol), in dry DMF (2.0 mL), triethylamine (1.5 mL, 11.1 mmol), bis(triphenylphosphine)palladium dichloride (5.4 mg, 0.0074 mmol), CuI (0.32 mg, 0.0018 mmol), and the suitable 1-alkyne (250 μL, 2.22 mmol) were added. The mixture was stirred under nitrogen at r. t. for 16 h, then the mixture was evaporated to dryness and the residue purified through silica gel flash column chromatography eluting with the suitable eluent. 

##### 2-Hexynyl-*N*^6^-(2-phenylethyl)adenosine (**10**)

Compound **10** was prepared from **8** by reaction with 1-hexyne. The mixture residue was purified through silica gel flash column chromatography eluting with CHCl_3_-MeOH (97:3) to acquire **10** as white solid. Yield: 38%; m.p.: 164–166 °C. ^1^H-NMR (DMSO-d_6_) δ: 0.90 (t, J = 7.2 Hz, 3H, CH_3_), 1.42 (m, 2H, CH_2_CH_3_), 1.52 (m, 2H, CH_2_CH_2_CH_3_), 2.42 (m, 2H, CH_2_-C≡C), 2.87 (m, 2H, CH_2_CH_2_NH), 3.33 (m, 2H, CH_2_CH_2_NH), 3.53 (m, 1H, HCH-5′), 3.64 (m, 1H, HCH-5′), 3.93 (q, J = 3.2 Hz, 1H, H-4′), 4.10 (m, 1H, H-3′), 4.50 (m, 1H, H-2′), 5.18 (m, 2H, 2 x OH), 5.44 (m, 1H, OH), 5.84 (d, J = 6.4 Hz, 1H, H-1′), 7.18 (m, 3H, H-Ph), 7.25 (m, 2H, H-Ph), 7.96 (m, 1H, NH), 8.37 (s, 1H, H-8). ESI-MS: positive mode m/z 451.9 [M+H]^+^, 473.9 [M+Na]^+^, 925.1 [2M+Na]^+^. Anal (C_24_H_29_N_5_O_4_) C, H, N.

##### 2-Phenylethynyl-*N*^6^-(2-phenethyl)adenosine (**11**)

Compound **11** was prepared from **8** by reaction with 1-phenylacetylene. The mixture residue was purified through silica gel flash column chromatography eluting with CHCl_3_-MeOH (96:4) to acquire **11** as white solid. Yield: 70%; m.p.: 230–232 °C. ^1^H-NMR (DMSO-d_6_) δ: 2.93 (m, 2H, CH_2_Ph), 3.57 (m, 2H, CH_2_NH), 3.65 (m, 1H, HCH-5′), 3.69 (m, 1H, HCH-5′), 3.95 (q, J = 3.2 Hz, 1H, H-4′), 4.12 (q, J = 3.2 Hz, 1H, H-3′), 4.53 (q, J = 5.2 Hz, 1H, H-2′), 5.21 (d, J = 4.8 Hz, 2H, 2 x OH), 5.49 (d, J = 6.0 Hz, 1H, OH), 5.89 (d, J = 6.0 Hz, 1H, H-1′), 7.46 (m, 3H, H-Ph), 7.62 (m, 2H, H-Ph), 7.19 (m, 3H, H-Ph), 7.28 (m, 2H, H-Ph), 8.1 (m, 1H, N^6^-H), 8.44 (s, 1H, H-8). ESI-MS: positive mode m/z 471.9 [M+H]+, 493.8 [M+Na]+, 965.0 [2M+Na]^+^. Anal (C_26_H_25_N_5_O_4_) C, H, N.

##### 2-Hexynyl-*N*^6^-(2,2-diphenylethyl)adenosine (**4**)

Compound **4** was prepared from **9** by reaction with 1-hexyne. The mixture residue was purified through silica gel flash column chromatography eluting with CHCl_3_-MeOH (97:3) to acquire **4** as white solid. Yield: 38%; m.p.: 164–166 °C. ^1^H NMR (DMSO-d6) δ: 0.93 (t, 3H, CH_3_), 1.47 (m, 2H, CH_2_CH_3_), 1.54 (m, 2H, CH_2_CH_2_CH_3_), 2.43 (m, 2H, CH_2_-C≡C), 3.53 (m, 1H, H-CH-5′), 3.62 (m, 1H, H-CH-5′), 3.92 (brs, 1H, H-4′), 4.06 (m, 1H, CH-Ph_2_), 4.09 (m, 1H, H-3′), 4.49 (m, 2H, CH_2_-NH), 4.55 (t, J = 7.6 Hz, 1H, H-2′), 5,17 (m, 2H, 2 x OH), 5.42 (d, J = 6 Hz, 1H, OH), 5.82 (d, J = 6 Hz, 1H, H-1′), 7,17 (m, 2H, H-Ph), 7.28 (m, 8H, H-Ph), 7.89 (app s, 1H, NH), 8.32 (s, 1H, H-8). ESI-MS: positive mode m/z 528.0 [M+H]^+^, 549.9 [M+Na]^+^. Anal (C_30_H_33_N_5_O_4_) C, H, N.

##### *N*^6^-(2,2-Diphenylethyl)-2-phenylethynyladenosine (**12**)

Compound **12** was obtained from **5** by reaction with phenylacetylene. The desired compound was obtained after purification through silica gel flash column chromatography eluting with DCM-MeOH (94: 6) as a yellow solid, yield: 51%; m.p.: 121–125 °C. ^1^H-NMR: (DMSO-d6) δ: 3.64 (m, 3H, H-5′), 3.97 (s, 1H, H-4′), 4.13 (s, 2H, NH-CH_2_), 4.55 (d, 2H, J = 4.5 Hz, H-3′), 4.61 (d, 1H, J = 7.5 Hz, H-2′), 5.22 (brt, 2H, OH), 5.47 (d, 1H, J = 5.5 Hz, OH), 5.89 (d, 1H, J = 5.5 Hz, H-1′), 7.59 (m, 15H, H-Ph), 8.00 (s, 1H, NH), 8.41 (s, 1H, H-8). ESI-MS: positive m/z 547.9 [M+H]^+^, 569.8 [M+Na]^+^. Anal (C_32_H_29_N_5_O_4_) C, H, N.

#### 3.1.8. Synthesis of 2,6-Dichloro-2′,3′,5′-tri-O-acetyl-(β-D-ribofuranosyl)purine (**14**)

Compound **6** (1.0 g, 2.3 mmol) was dissolved in dry DCM (40 mL). Then, SbCl_3_ (746.5 mg, 3.27 mmol) and isoamyl nitrite (1.4 mL, 9.89 mmol) were added to the solution and it was left to react at 0 °C for 5 h. After this time, volatiles were removed under vacuum, the mixture was then extracted with DCM, the organic layer collected and dried over Na_2_SO_4_. The solvent was filtered and evaporated and the mixture was made in to slurry and purified by flash chromatography eluting with DCM/MeOH (99.5:0.5). Compound **14** was obtained as a white solid after crystallization with MeOH. Yield: 73%; m.p. 132–134 °C; ^1^H-NMR (DMSO-d6) δ: 2.00 (s, 3H, CH_3_), 2.04 (s, 3H, CH_3_), 2.11 (s, 3H, CH_3_), 4.29 (d, 2H, J = 3.4 Hz, CH-5′), 4.31 (d, 1H, J = 2.4 Hz, H-4′), 5.63 (t, 1H, J = 4.0 Hz, H-3′), 5.91 (t, 1H, J = 5.4 Hz, H-2′), 6.32 (d, 1H, J = 5.5 Hz, H-1′), 8.92 (s, 1H, H-8). ESI-MS: positive mode m/z 468.7 [M+Na]^+^. Anal C_16_H_16_Cl_2_N_4_O_7_) C, H, N.

#### 3.1.9. General Procedure for the Synthesis of 2-Chloro-*N*^6^-substituted adenosines **15** and **16**

Compound **14** (120 mg, 0.26 mmol) was added, in turn, to a solution of phenylethylamine (0.1 mL, 0.79 mmol) and Et_3_N (0.1 mL) in absolute ethanol (10 mL) or a solution of 2,2-diphenylethylamine (122 mg, 0.79 mmol) and Et_3_N (0.1 mL) in absolute ethanol (10 mL). The reaction was left under stirring 15 h at r. t., then, NH_3_/MeOH (10 mL) was added and the reaction left under stirring 1 h. Volatiles were removed under vacuum, the mixture made into slurry and purified by flash chromatography eluting with the suitable solvent.

#### 3.1.10. 2-Chloro-*N*^6^-(2-phenylethyl)adenosine (**15**)

Compound **15** was obtained by reaction of **14** with 2-phenethylamine. The final compound was obtained after chromatography eluting with CH_2_Cl_2_-MeOH (97:3) and crystallization with EtOAc/n-Hex/diethyl ether as a white powder. Yield: 47%; m.p.: 153–155 °C. ^1^H-NMR (DMSO-d6) δ: 2.90 (t, 2H, CH_2_-Ph), 3.59 (m, 3H, H-5′), 3.94 (d, 1H, J = 4.5 Hz, H-4′), 4.05 (s, 1H, NH-CH_2_), 4.11 (d, 1H, J = 5.0 Hz, H-3′), 4.49 (m, 1H, H-2′), 5.05 (d, 1H, J = 6.5 Hz, OH), 5.22 (d, 1H, J = 4.5 Hz, OH), 5.48 (d, 1H, J = 8.0 Hz, OH), 5.80 (d, 1H, J = 7.0 Hz, N-H’), 7.32 (m, 5H, Ph), 8.37 (s, 1H, N-H-8), 8.45 (d, 1H, J = 7.5 Hz, N-H). ESI-MS: positive mode m/z 427.8 [M+Na]^+^. Anal (C_18_H_20_ClN_5_O_4_) C, H, N.

#### 3.1.11. 2-Chloro-*N*^6^-(2,2-diphenylethyl)-adenosine (**16**)

Compound **16** was obtained by reaction of 14 with 2,2-diphenylethylamine. The final compound was obtained after chromatography eluting with CH_2_Cl_2/_MeOH (95:5) and crystallization with ethyl acetate/n-Hex/diethyl ether as white powder. Yield: 67%; m.p.: 110–113 °C; ^1^H-NMR (DMSO-d_6_) δ: 3.55 (m, 3H, H-5′), 3.94 (t, 1H, H-4′), 3.96 (brs, 2H, CH_2_-Ph), 4.1 (m, 2H, H-3′), 4.48 (brs, 1H, NH-CH_2_), 4.52 (m, 1H, H-2′), 4.55 (d, 1H, J = 5 Hz, OH), 5.06 (d, 1H, J = 5 Hz, OH), 5.21 (d, 1H, J = 5 Hz, OH), 5.81 (d, 1H, J = 5 Hz, H-1′), 7.19 (m, 10H, Ph), 8.33 (s, 1H, H-8), 8.51 (s, 1H, NH). ESI-MS: positive mode m/z 482.8 [M+H]^+^, 504.1 [M+Na]^+^. Anal (C_24_H_24_ClN_5_O_4_) C, H, N.

### 3.2. Biological Assays at Human Adenosine Receptors

#### 3.2.1. Cell Culture

Chinese hamster ovary (CHO) cells stably expressing human ARs were grown adherently and maintained in Dulbecco’s Modified Eagles Medium with nutrient mixture F12 (DMEM/F12), supplemented with 10% Fetal Bovine Serum (FBS), 100 U/mL penicillin, 100 µg/mL streptomycin, 2.5 µg/mL amphotericin, 1mM Sodium pyruvate and 0.1 mg/mL Geneticin (G418) at 37 °C and aerated with 5% CO_2_: 95% O_2_.

#### 3.2.2. Membrane Preparation

All pharmacological methods followed the procedures as described earlier [22]. In brief, membranes for radioligand binding were prepared from CHO cells stably transfected with human adenosine receptor subtypes through two centrifugations at different speeds. The first low-speed (1000 g) centrifugation allowed the removing of cell fragments and nuclei, while the second, performed at high speed (100,000 g), allowed the precipitation of the crude membrane fractions.

The resulting membrane pellet was resuspended in the buffer used for the respective binding experiments, frozen in liquid nitrogen and stored in aliquots at −80 °C.

#### 3.2.3. Binding Assay 

The binding affinity of the novel compounds was evaluated using radioligand competition experiments in CHO cells stably expressing hA_1_AR, hA_2A_AR, and hA_3_AR subtypes. The radioligands used were 1.0 nM [^3^H]CCPA (K_D_ = 1.1 nM) for hA_1_, 10 nM [^3^H]NECA (K_D_ = 20 nM) for hA_2A_, and 1.0 nM [^3^H]HEMADO (K_D_ = 1.5 nM) for hA_3_ receptors. Results were expressed as K_i_ values (dissociation constants), which were calculated with the program GraphPad (GraphPAD Software, San Diego, CA, USA). Each concentration was tested three–five times in duplicate and the values are given as the mean ± standard error (S.E.).

The potency of compounds at the hA_2B_ receptor (expressed on CHO cells) was determined through GloSensor cAMP Assay.

#### 3.2.4. Functional Agonism or Antagonism at A_2B_ or A_3_ ARs in GloSensor cAMP Assay

CHO cells stable expressing A_2B_ or A_3_ARs and the plasmid encoding the biosensor were used to study functional agonism and antagonism of understudy ligands. The desired cell number was incubated in equilibration medium containing a 3% *v*/*v* GloSensor cAMP reagent stock solution, 10% FBS and 87% of CO_2_ independent media. After 2 h of incubation, cells were dispensed in wells of 384 well plate and when a steady-state basal signal was obtained the agonism profile was studied, adding to wells the reference agonist NECA or compounds at different concentrations. In the case of the G_i_ coupled receptor A_3_AR Forskolin (FSK) 10 μM was added 10 min after the agonists and various luminescence readings were performed at different incubation times.

The antagonist profile of the compounds was evaluated by assessing their ability to counteract an agonist-induced increase or decrease (A_2B_ and A_3_ARs, respectively) of cAMP accumulation [22]. The cells were incubated with different antagonist concentrations and then treated with a fixed dose of NECA (10 μM for A_2B_AR and 1 μM for A_3_AR). FSK 10 μM was added 10 min after the agonists and various luminescence reads were performed at different incubation times.

Responses were expressed as percentage of the maximal relative luminescence units (RLU) and concentration–response curves were fitted by a nonlinear regression with the Prism program (GraphPAD Software, San Diego, CA, USA). The agonist or antagonist profile of compounds was expressed as EC_50_ or IC_50_, respectively [30]. Each concentration was tested three–five times in duplicate and the values are given as the mean ± S.E.

### 3.3. Biological Studies on Cancer Cell Line

#### 3.3.1. Cell Lines 

The PC3 prostate cancer cell line used is from Type Culture Collection, ATCC, (Rockville, MD, USA). Cells were cultured in Dulbecco’s modified Eagle’s medium (DMEM) supplemented with 10% fetal bovine serum, 2 mM L-glutamine, 100 IU/mL penicillin, 100 μg streptomycin at 37 °C in a humidified incubator containing 5% CO_2_.

#### 3.3.2. Reagents

DMSO (used as vehicle), sulforhodamine B (SRB), trichloroacetic acid (TCA), and acetic acid were from Sigma Aldrich (Sant Louis, MO, USA). 

#### 3.3.3. Cell Growth Inhibition Assay

To determine the effects of compounds **4**, **5, 8–13, 15, 16**, and Cl-IB-MECA on PC3 cell line, an SRB assay was performed. Cells were seeded at 4 × 10^3^ cells/well in a 96-well microplate. Twenty-four hours later, cultures were treated with increasing concentrations of compounds, 1, 10, 25, 50, 100 μm, for 48 h at 37 °C in a 5% CO_2_ atmosphere and 95% relative humidity. At the same time (t = 0), and after drug treatments, 100 μL of 10% (*w*/*v*) TCA were added to each well, incubated for 1 h at 4 °C, washed with deionized water, and dried at room temperature. One hundred microliters of SRB solution were added to each well, incubated for 10 min at room temperature, rinsed four times with 1% (*v*/*v*) acetic acid, and allowed to dry at room temperature. Finally, 100 μL of 10 mM Tris base solution (pH 10.5) was added to each well, and the absorbance was measured at 515 nm in a microplate reader (BioTek Instruments, Winooski, VT, USA). The absorbance at t = 0 was compared with the absorbance at the end of the experiment to determine cell growth in treated cells compared with control cells. The antitumor activity was estimated by measurements of three parameters: Growth Inhibition 50 (GI_50_), the drug concentration (µM) required to inhibit 50% net of cell growth; Total Growth Inhibition (TGI), the drug concentration (µM) required to inhibit 100% of cell growth; and Lethal Concentration 50 (LC_50_), the drug concentration (µM) required to kill 50% of the initial cell number. LC_50_, GI_50_ and TGI values are shown as mean ± standard deviation (SD) of three different experiments calculated using GraphPad Prism 5.0 (GraphPad Software, San Diego, CA, USA).

#### 3.3.4. Statistical Analysis

The statistical significance was determined by Student’s t-test by using, as control, the reference compound 2-Cl-IB-MECA. * *p* < 0.05.

## 4. Conclusions

Novel di-substituted Ado derivatives bearing a *N*^6^-phenethyl or a more steric hindered *N*^6^-(2,2-diphenylethyl) group combined with halogens, alkynes or a phenylethylthio chain in 2-position were synthesized and tested in radioligand binding assays at A_1_, A_2A_, and A_3_ ARs. Selected compounds were also tested in a functional assay at A_2B_ARs and, in most cases, were found not able to activate the receptor at concentrations up to 30 µM. In the binding studies, all the compounds were found to be A_3_AR ligands with high affinity (Ki in the nM and sub-nM range) and different degree of selectivity. In general, compounds bearing a 2-phenethylamino group in *N*^6^-position resulted endowed with higher affinity and selectivity than the corresponding analogues bearing a more hindered 2,2-diphenylethyl group in the same position. Furthermore, in the PC3 prostate cancer cell line, all the compounds concentration-dependently reduced the cell growth and most of them showed a significant inhibitory effect on proliferation and a pronounced cytotoxic activity comparable to that of Cl-IB-MECA. In particular, the ligands with the best cytostatic properties were those bearing a *N*^6^-(2,2-diphenylethyl) group, indicating that the antitumor activity is not closely related to the affinity for the A_3_AR subtype. Finally, functional cAMP assays demonstrated that compounds endowed with the best cytotoxic activity behave as A_3_AR antagonists, confirming the hypothesis that other cellular mechanisms are involved in the anticancer properties of these A_3_AR ligands. Therefore, further experiments will be needed to explain the precise pathways responsible for their anticancer effects. 

## Figures and Tables

**Table 1:**
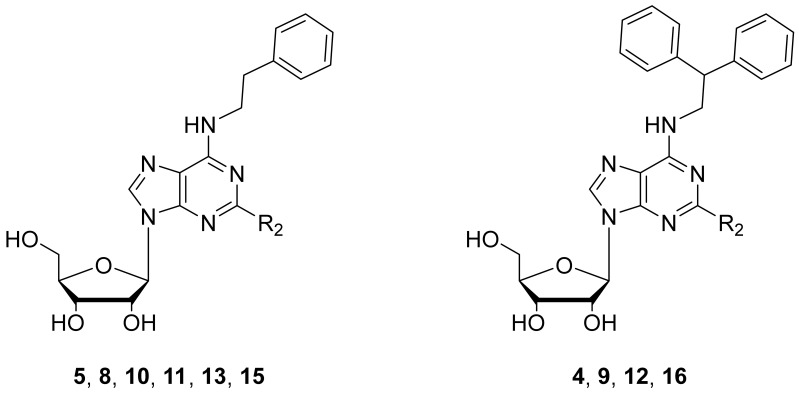
Affinity (Ki, nM) of compounds **4**, **5**, **8–13**, **15**, and **16** in radioligand binding assays at human A_1_, A_2A_, and A_3_ ARs subtypes.

Cmp	R_2_	hA_1_R ^a^ (K_i_ nM)	hA_2A_R ^b^ (K_i_ nM)	hA_3_R ^c^ (K_i_ nM)	A_1_/A_3_	A_2A_/A_3_
**Cl-IB-MECA** [24]		1240	5360	1.4	886	3829
**5** [18]	NHCH_2_CH_2_Ph	357	1368	0.33	1082	4145
**8**	I	144 ± 32	4119 ± 998	5 ± 1.2	29	824
**15**	Cl	1.64 ± 0.37	660 ± 129	0.024 ± 0.005	68	27,500
**13**	SCH_2_CH_2_Ph	263 ± 19	3359 ± 752	30 ± 7.1	9	112
**10**	C≡C(CH_2_)_3_CH_3_	129 ± 8.2	146 ± 48	1.5 ± 0.3	86	97
**11**	C≡CPh	809 ± 117	2983 ± 271	3.8 ± 0.6	213	785
**9**	I	182 ± 25.4	1243 ± 253.1	11 ± 1.8	17	113
**16**	Cl	0.76 ± 0.2	266 ± 35	0.13 ± 0.01	6	2046
**4** [18]	C≡C(CH_2_)_3_CH_3_	984	153	27	36	6
**12**	C≡CPh	211 ± 53	450 ± 84	106 ± 25	2	4

^a^ Displacement of specific [^3^H]CCPA binding in membranes from CHO cells, stably transfected with human recombinant A_1_AR. ^b^ Displacement of specific [^3^H]NECA binding in membranes from CHO cells, stably transfected with human recombinant A_2A_AR. ^c^ Displacement of specific [^3^H]HEMADO binding in membranes from CHO cells, stably transfected with human recombinant A_3_AR. Data (*n* = 3–5) are expressed as means ± standard errors.

**Table 2:**
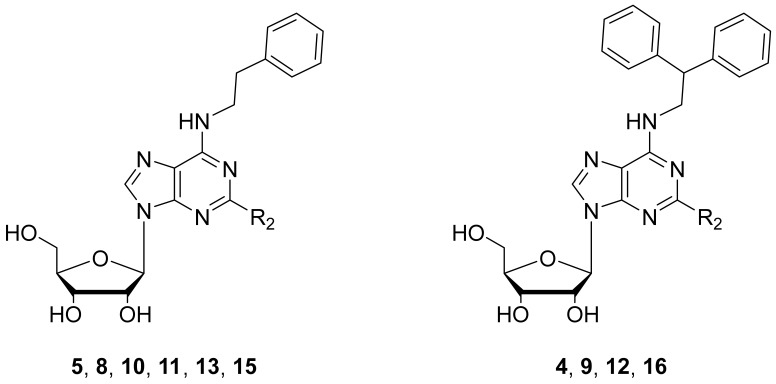
Cytostatic and cytotoxic activity of compounds **4**, **5**, **8–13**, **15**, and **16** determined with the sulforhodamine B assay. The evaluated parameters GI_50_, TGI, and LC_50_ are reported in comparison of A_3_AR affinity of tested compounds.

Cpd	R_2_	GI_50_ (μM)	TGI (μM)	LC_50_ (μM)	A_3_AR (nM)
Cl-IB-MECA		18 ± 0.4	44 ± 3.3	110 ± 8.4	1.4
**5**	NHCH_2_CH_2_Ph	237 ± 9.4	>500	>500	0.33
**8**	I	42 ± 2.7	113 ± 6.7	301 ± 13.4	5.0
**15**	Cl	51 ± 5.2	262 ± 8.3	>500	0.024
**13**	SCH_2_CH_2_Ph	41 ± 4.2	>500	>500	30
**10**	C≡C(CH_2_)_3_CH_3_	13 ± 1.6	77 ± 2.9	452 ± 10.5	1.5
**11**	C≡CPh	2.5 ± 0.8	19 ± 6.1	151 ± 8.9	3.8
**9**	I	24 ± 1.2	48 ± 3.5	94 ± 3.5	11
**16**	Cl	35 ± 3.1	94 ± 4.1	253 ± 11.5	0.13
**4**	C≡C(CH_2_)_3_CH_3_	16 ± 0.5	35 ± 1.5	80 ± 9.8	27
**12**	C≡CPh	14 ± 0.9	29 ± 4.9	59 ± 10.1	106

LC_50_, GI_50_ and TGI values are shown as mean ± standard deviation (SD) of three different experiments calculated using GraphPad Prism 5.0 (GraphPad Software, San Diego, CA, USA).

**Table 3:** Activity of **4**, **10–12**, and **15**, compared to Cl-IBMECA, in a cAMP accumulation assay at the human A_3_AR stably transfected in CHO cells.

Cpd	A_3_AR CHO Cells	
	EC_50,_ nM	IC_50_, nM
Cl-IB-MECA	2.8 ± 1.4	
**15**	14 ± 3.4	
**10**		31 ± 6
**11**		79 ± 15
**4**		380 ± 80
**12**		153 ± 12

The EC_50_ or IC_50_ values are calculated from concentration–response curves fitted by a nonlinear regression with the Prism program (GraphPAD Software, San Diego, CA, USA). Each concentration was tested three–five times in duplicate and the values are given as the mean ± S.E.

## Data Availability

Data is contained within the article and supplementary material.

## Supplementary Materials

The following are available online at https://www.mdpi.com/article/10.3390/ph15020164/s1, Table S1: Elemental analysis data of evaluated compounds **4**, **5**, **8–13**, **15**, **16**; Figure S1: Concentration-response curves of the sulforhodamine B (SRB) assay; Figure S2: Concentration–response curves of considered compounds in GloSensor cAMP functional assay performed at CHO cells stable transfected with hA_3_AR.
