# Supplementary material for: A3 Adenosine Receptor Antagonists with Nucleoside Structures and Their Anticancer Activity
Source: Pharmaceuticals (Basel). 2022 Jan 28;15(2):164. doi: 10.3390/ph15020164 (PMC8879107; doi:10.3390/ph15020164)
Supplement: Supplementary file 1 [file pharmaceuticals-15-00164-s001.zip › pharmaceuticals-1557769-supplementary.pdf]

A_3 adenosine receptor antagonists with nucleoside structures and their anticancer activity

Andrea Spinaci [1], Michela Buccioni [1], Diego Dal Ben [1], Federica Maggi [2,3], Gabriella Marucci [1], Beatrice Francucci [1], Giorgio Santoni [2], Catia Lambertucci [1*], Rosaria Volpini [1*]

[1]Medicinal Chemistry Unit, School of Pharmacy, University of Camerino, 62032 Camerino (MC), Italy; [2]Experimental Medicine Section, School of Pharmacy, University of Camerino, 62032 Camerino (MC), Italy; [3]Department of Molecular Medicine, Sapienza University, 00185 Rome, Italy;

*Correspondence: rosaria.volpini@unicam.it; Tel.: +39-0737402278

Supplementary materials

Contents

S1. Chemistry……………………………………………………………………….......... 1
S.1.1. Elemental analysis data …………………………………………………………………… 1
S2. Biological Evaluation………………………………………………………………… 2
S.2.1. Concentration-response curves sulforhodamine B (SRB) assay ..…………………… 2
S.2.2. Concentration-response curves adenylyl-cyclase assay in hA_3AR ……………….. 7

S1. Chemistry

S.1.1. Elemental analysis data of final compounds: elemental analyses were determined on Fisons Instruments Model EA 1108 CHNS-O model analyser and are within 0.4% of theoretical values. Purity of the compounds is ≥ 98% according to elemental analysis data.

Table S1. Elemental analysis data of evaluated compounds **4**, **5**, **8-13**, **15**, **16**.

Cpd	Mol. Form.	Elemental analysis calc.			Elemental Analysis found		
		C	H	N	C	H	N
**4**	$C_{30}H_{33}N_5O_4$	68.29	6.30	13.27	68.33	6.32	13.01
**5**	$C_{26}H_{30}N_6O_4$	63.66	6.16	17.13	63.78	6.18	16.89
**8**	$C_{18}H_{20}IN_5O_4$	43.47	4.05	14.08	43.66	4.05	13.95
**9**	$C_{24}H_{24}IN_5O_4$	50.27	4.22	12.21	50.42	4.25	12.01
**10**	$C_{24}H_{29}N_5O_4$	63.84	6.47	15.51	63.99	6.49	15.62
**11**	$C_{26}H_{25}N_5O_4$	66.23	5.34	14.85	66.31	5.34	14.75
**12**	$C_{32}H_{29}N_5O_4$	70.19	5.34	12.79	70.37	5.37	12.67
**13**	$C_{26}H_{29}N_5O_4S$	61.52	5.76	13.80	61.59	5.80	13.69
**15**	$C_{18}H_{20}ClN_5O_4$	53.27	4.97	17.26	53.31	5.00	16.92
**16**	$C_{24}H_{24}ClN_5O_4$	59.81	5.02	14.53	59.97	5.03	14.22

S2. Biological Evaluation

S.2.1. Concentration-response curves of the sulforhodamine B (SRB) assay.

Concentration-response curves of the sulforhodamine B (SRB) assay in PC3 prostate cell line have been obtained through evaluation of cell viability at the concentrations 1, 10, 25, 50, and 100 µM for 48 h at 37 °C of compounds **4**, **5**, **8-13**, **15**, and **16**. From the curve three parameters have been measured: Growth Inhibition 50 (GI_{50}), the compound concentration (µM) required to inhibit 50% net of cell growth; Total Growth Inhibition (TGI), the compound concentration (µM) required to inhibit 100% of cell growth; Lethal Concentration 50 (LC_{50}), the compound concentration (µM) required to kill 50% of the initial cell number. LC_{50}, GI_{50} and TGI values are shown as mean ± standard deviation (SD) of three different experiments calculated using GraphPad Prism 5.0 (GraphPad Software, San Diego, CA, USA). The statistical significance was determined by Student's t-test by using as control the reference compound Cl-IB-MECA. *$p<0.05$.

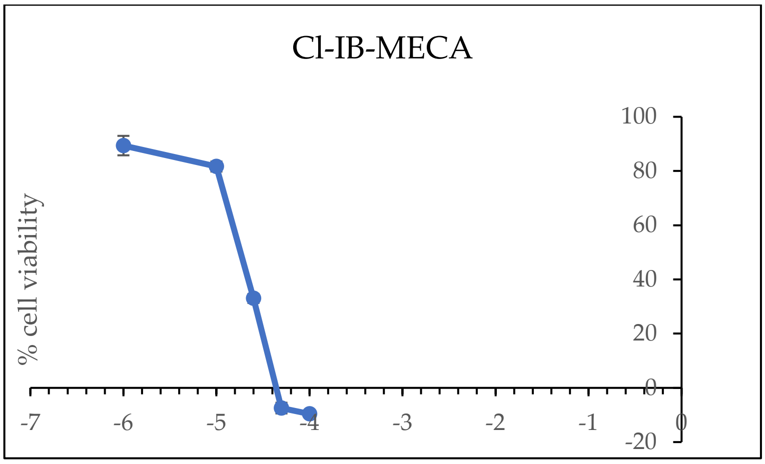

	**GI_{50}**	**TGI**	**LC_{50}**
Cl-IB-MECA	18 µM	44 µM	110 µM

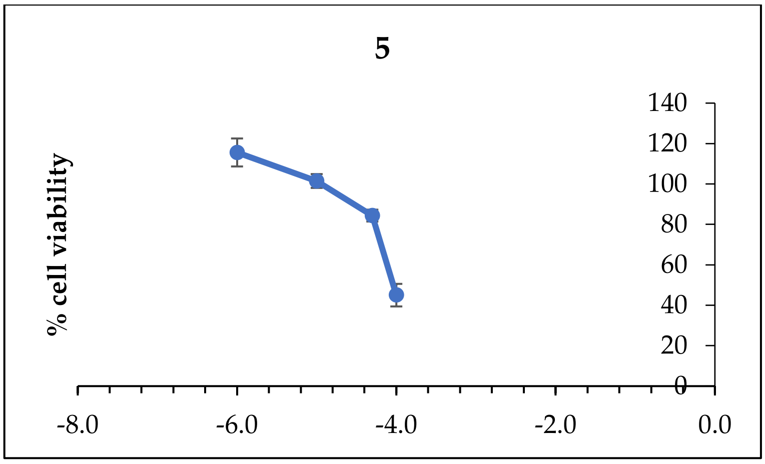

	**GI_{50}**	**TGI**	**LC_{50}**
**5**	237 µM	> 500 µM	> 500 µM

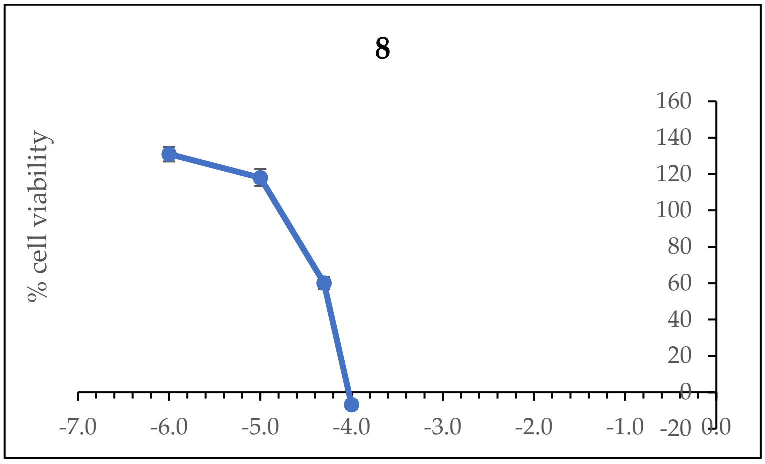

	GI50	TGI	LC50
**8**	42 μM	113 μM	301 μM

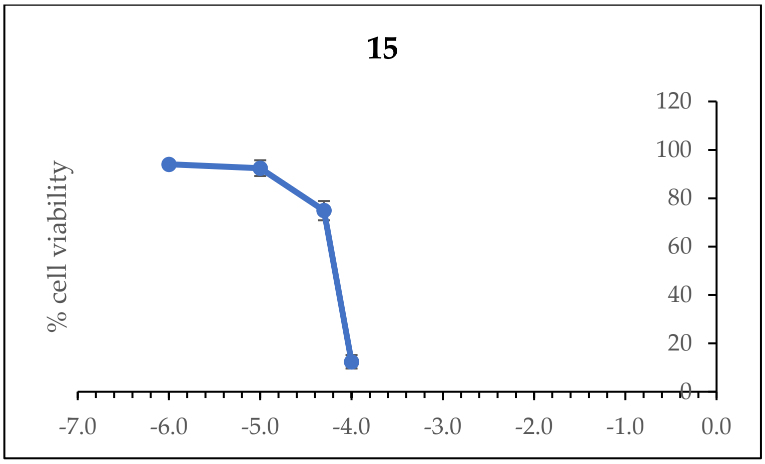

	GI50	TGI	LC50
**15**	51 μM	262 μM	> 500 μM

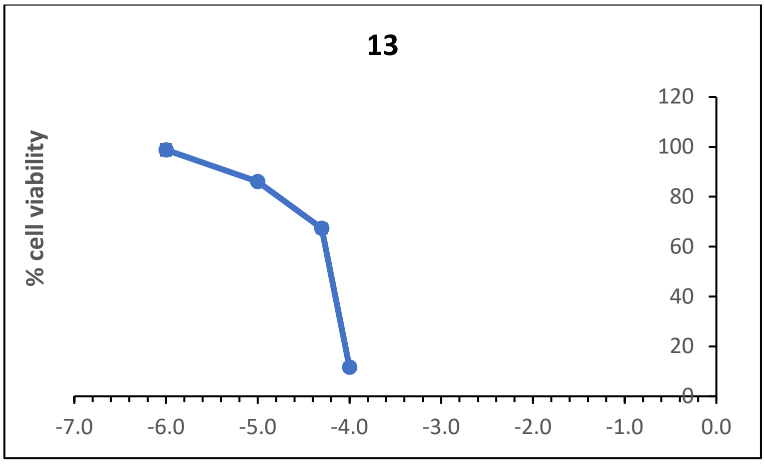

	GI50	TGI	LC50
**13**	41 μM	> 500 μM	> 500 μM

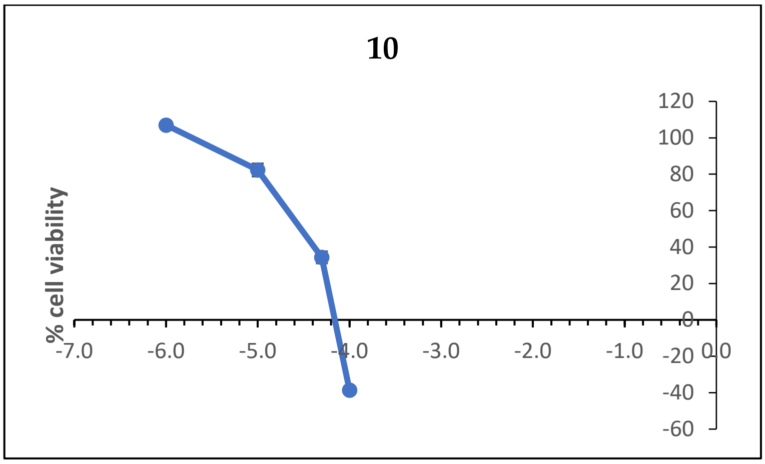

	GI50	TGI	LC50
**10**	13 µM	77 µM	452 µM

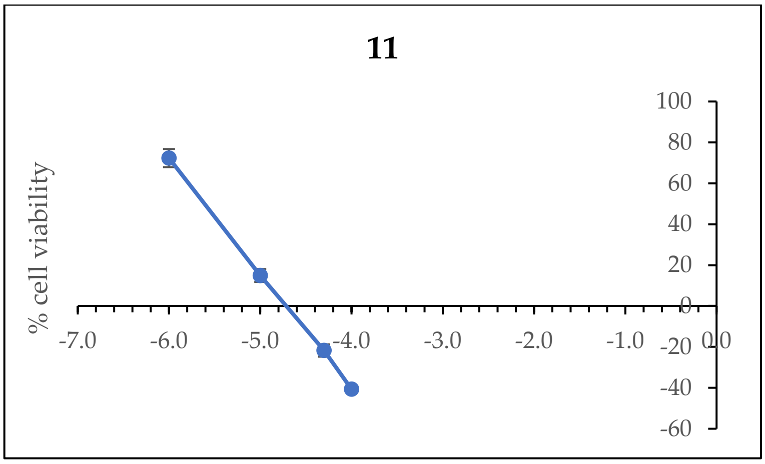

	GI50	TGI	LC50
**11**	2.5 µM	19 µM	151 µM

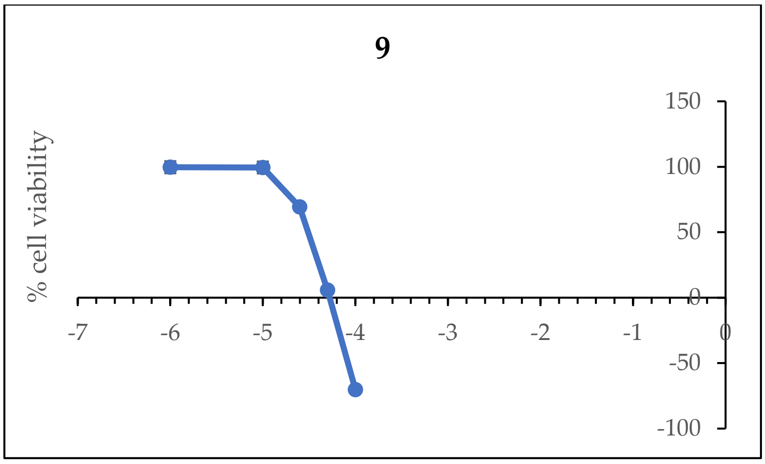

	GI50	TGI	LC50
**9**	24 µM	48 µM	94 µM

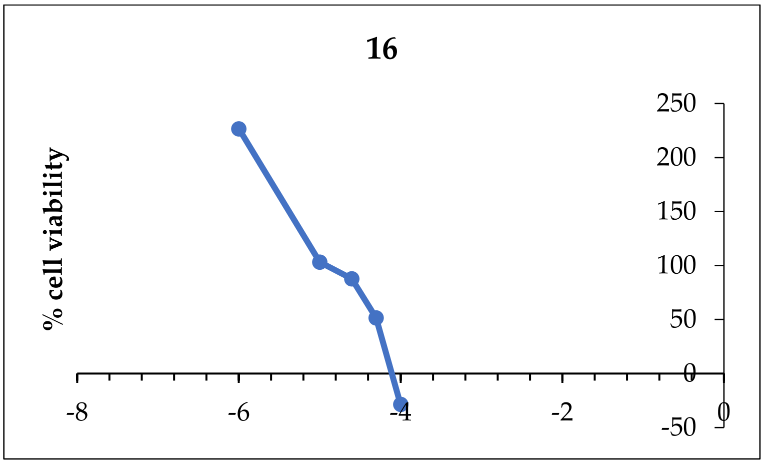

	GI50	TGI	LC50
**16**	35 µM	94 µM	253 µM

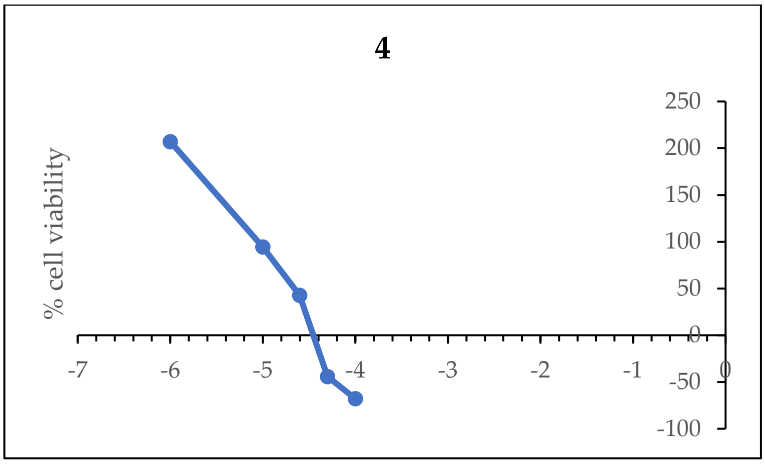

	GI50	TGI	LC50
**4**	16 µM	35 µM	80 µM

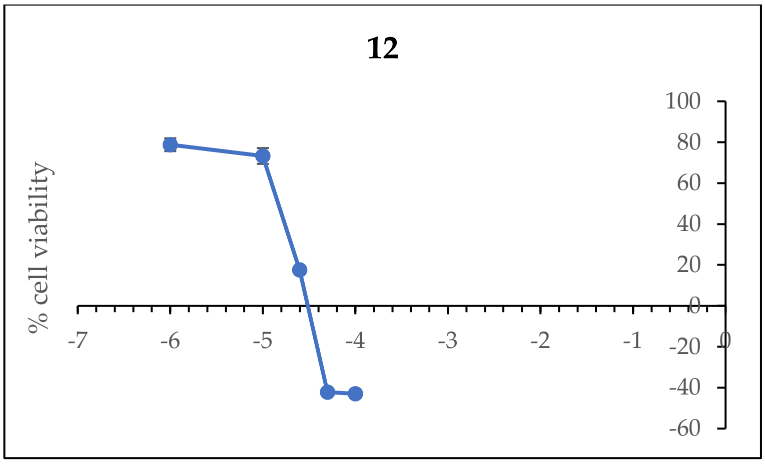

	GI50	TGI	LC50
**12**	14 µM	29 µM	59 µM

Figure S1. Concentration-response curves of the sulforhodamine B (SRB) assay in PC3 prostate cell line have been obtained through evaluation of cell viability at the concentrations 1, 10, 25, 50, and 100 µM for 48 h at 37 °C of compounds **4**, **5**, **8-13**, **15**, and **16**. LC_{50}, GI_{50} and TGI values are shown as mean ± standard deviation (SD) of three different experiments calculated using GraphPad Prism 5.0 (GraphPad Software, San Diego, CA, USA). The statistical significance was determined by Student's t-test by using as control the reference compound Cl-IB-MECA. *p<0.05.

S.2.2. Concentration-response curves of adenylyl-cyclase assay in hA_3AR

For each ligand the ability to inhibit forskolin stimulated cAMP production through the human A_3AR was assessed (agonists); for those compounds that did not affect forskolin-induced cAMP production, the ability to counteract an agonist-induced decrease of cAMP accumulation was evaluated (antagonists).

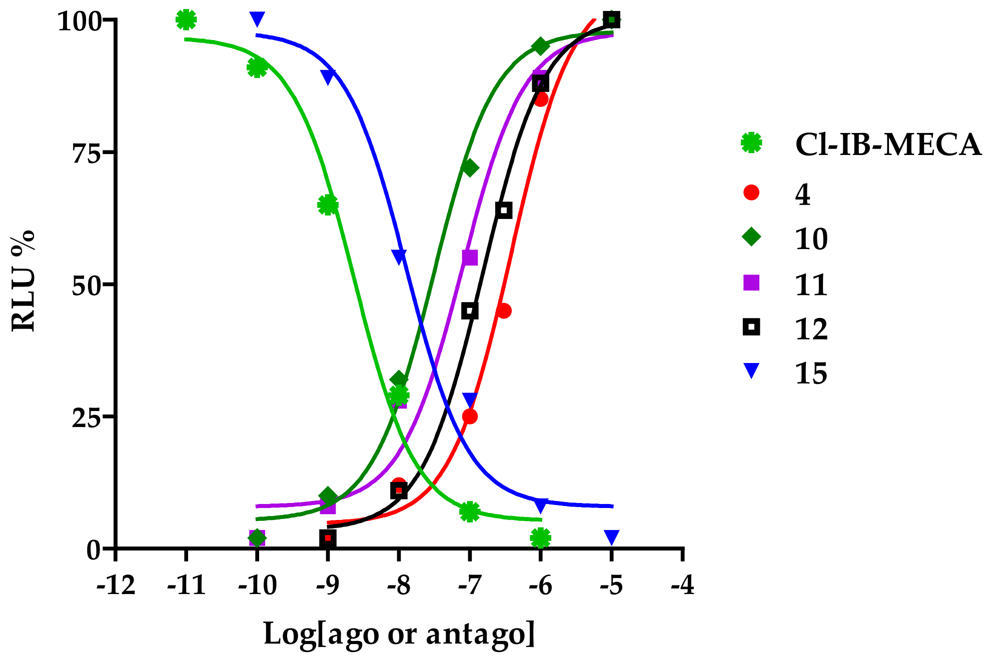

Figure S2. Concentration–response curves of considered compounds in GloSensor cAMP functional assay performed at CHO cells stable transfected with hA_3AR. Each point represents the mean of three-five experiments performed in duplicate with a maximum SE lower than ± 10. (RLU = Relative Luminescence Units)
